# Regulation of T Cell Responses by Ionic Salt Signals

**DOI:** 10.3390/cells10092365

**Published:** 2021-09-09

**Authors:** Christina E. Zielinski

**Affiliations:** 1 Department of Infection Immunology, Leibniz Institute for Natural Product Research and Infection Biology, Hans-Knoell-Institute, 07745 Jena, Germany; christina.zielinski@leibniz-hki.de; 2 Department of Biological Sciences, Friedrich Schiller-University, 07743 Jena, Germany

**Keywords:** T cells, immune regulation, cytokines, autoimmunity, allergy, sodium chloride

## Abstract

T helper cell responses are tailored to their respective antigens and adapted to their specific tissue microenvironment. While a great proportion of T cells acquire a resident identity, a significant proportion of T cells continue circulating, thus encountering changing microenvironmental signals during immune surveillance. One signal, which has previously been largely overlooked, is sodium chloride. It has been proposed to have potent effects on T cell responses in the context of autoimmune, allergic and infectious tissue inflammation in mouse models and humans. Sodium chloride is stringently regulated in the blood by the kidneys but displays differential deposition patterns in peripheral tissues. Sodium chloride accumulation might furthermore be regulated by dietary intake and thus by intentional behavior. Together, these results make sodium chloride an interesting but still controversial signal for immune modulation. Its downstream cellular activities represent a potential therapeutic target given its effects on T cell cytokine production. In this review article, we provide an overview and critical evaluation of the impact of this ionic signal on T helper cell polarization and T helper cell effector functions. In addition, the impact of sodium chloride from the tissue microenvironment is assessed for human health and disease and for its therapeutic potential.

## 1. Introduction

T cell responses are adapted to their respective cognate antigen and display a high degree of heterogeneity to meet the challenges of antigen elimination in varying tissue contexts. Upon an antigen encounter, conveyed through presentation by antigen-presenting cells, several polarizing signals educate naïve T cells to develop into mature effector and memory T cells with specialized functions in terms of cytokine production and migration [1]. The three classic signals are T cell receptor activation, costimulation and cytokines. Their differential integration in both quality and quantity gives rise to an increasing family of distinct T helper cell subsets. Th1 and Th2 cells emerged as early representatives of T helper cell subsets before the discovery of Th17 cells in 2007 sparked the quest for further identification and characterization of novel T helper cell subsets, such as Th22, Th-GMCSF and Th9 cells [2,3,4,5,6,7]. Polarizing cytokines in the context of antigen recognition and their downstream signaling pathways have been the primary focus for dissecting T helper cell diversification into their respective subsets. This approach has come with tremendous translational success since therapeutic blockade of polarizing cytokines abrogates pathogenic T helper cell functions in various disease activities. For example, the neutralization of IL-23, a Th17 cell-stabilizing cytokine, reduces pathogenic Th17 cell responses with previously unmatched therapeutic success in psoriasis as well as other chronic inflammatory diseases [8]. Likewise, IL4Ra blockade with the monoclonal antibody dupilumab efficiently abrogates the inflammatory activities of IL-14 and IL-13 in allergic diseases, such as asthma and atopic dermatitis [9,10]. More diverse signals from the surrounding tissue microenvironment can be integrated by T cells to tailor their functions to their acute contextual situation. Metabolites from host cells, as well as from commensal or pathogenic microorganisms [11], vitamins [12] and oxygen levels [13], can affect T cell fates. Additionally, these external cues, together with cytokines, can also act on fully differentiated effector T cells and memory T cells to modulate their properties and to even cause plastic shifts in their identity as a T helper cell subset.

One signal, which has previously come to attention as a novel immunoregulator, is sodium chloride. Despite its well-established role in human health and disease and the sophisticated control mechanisms exerted by the kidneys to maintain electrolyte homeostasis, its impact on the immune system has been largely ignored for a long time despite early evidence for osmotic regulation of cytokine synthesis [14]. Accumulating data, however, suggest a significant impact of sodium chloride on various cell types as discussed previously [15] and, in particular, on T cell responses in both homeostasis and pathological conditions as discussed herein.

## 2. Ionic Signals in the Tissue Microenvironment

**Figure 1:**
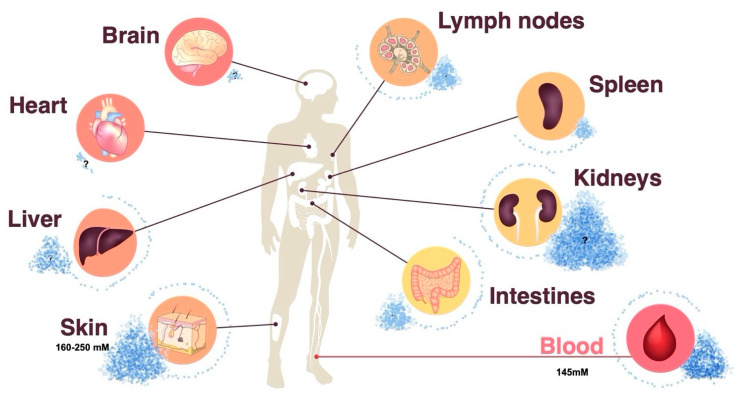
Differential distribution of sodium chloride in human organs. The skin can dynamically store sodium chloride, and thus exceed systemic sodium chloride concentrations in the peripheral blood. Dietary salt intake contributes to sodium chloride accumulation in the skin and possibly other peripheral organs. Neutron activation analysis represents a very sensitive method for the quantification of tissue sodium chloride concentrations. Question marks indicate that sodium chloride concentrations in the indicated organs remain to be established.

Sodium chloride has been largely overlooked as an immunoregulatory factor in the past because its concentration in body fluids is tightly controlled and stably maintained by the kidneys [16]. While this is supported by blood monitoring, peripheral tissue sodium concentrations have been previously shown to dynamically adapt to dietary changes in sodium chloride intake and to display differential sodium concentrations depending on the type of tissue, age, sex and inflammatory state [15,17,18]. This evidence implies a heterogeneous landscape of sodium chloride concentrations throughout the human body (Figure 1). Since sodium chloride dissociates into positively charged Na^+^ and negatively charged Cl^–^ ions in body tissues, the detection of sodium is complicated by its noncovalent binding to negatively charged tissue components, such as glycosaminoglycans and hyaluronic acid. Several qualitative and quantitative analysis strategies have been employed to assess salt concentrations in an organic matrix. Neutron activation analysis is a very sensitive quantitative technology. It is based on the neutron irradiation of the sample under consideration in a nuclear reactor leading to the production of radioactive isotopes emitting gamma quanta of characteristic energy. The intensity of the characteristic gamma radiation allows the determination of the activity (given in Bq) of the emitter [19,20]. The mass elements O, H, C and N in organic tissue are hardly activated by thermal neutron irradiation, whereas Na exhibits a high activation cross section, in other words, the absorption probability for thermal neutrons. The activation product Na-24 emits two clearly measurable gamma quanta for its identification and allows for unparalleled precise sodium quantification [19]. Alternative technologies, such as sodium (^23^Na) magnetic resonance (MR) imaging, also serve the purpose of sodium visualization and quantification in tissues. The intricacies and limitations of this method, which is gaining increasing popularity in clinical settings, are reviewed elsewhere [21]. Lastly, chemical methods, such as inductively coupled plasma-optical emission spectrometry, allow for sodium quantification in multi-step processes [22].

In particular, the skin has been demonstrated to serve as an interstitial sodium reservoir [23,24,25]. The mechanisms for differential sodium storage in peripheral tissues remain largely elusive. Therapeutic inhibition of glucose and sodium reabsorption from urine with inhibitors of sodium glucose cotransporter 2 (SGLT2) results in a reduction in cutaneous sodium concentrations. This effect has also been observed after diuretic treatment in patients with heart failure [15,26]. The enhanced sulfation of glycosaminoglycans in rats has been shown to serve as a negatively charged capacitor of positively charged sodium ions [25,27]. Dietary sodium chloride accumulation is associated with increased chondroitin synthase mRNA content in the skin. The extent of glycosaminoglycan (GAG) chain polymerization may therefore regulate sodium storage in the skin or other tissues [22,28]. Increased lymph flow following lymph capillary hyperplasia increases salt clearance from cutaneous stores. Interestingly, macrophages have been reported to participate in this process through their ability to produce vascular endothelial growth factor C (VEGFC) in response to salt [15,24].

Circulating immune cells are faced with a continuously changing sodium microenvironment throughout their system-wide immune surveillance under physiological conditions [29]. Under pathological conditions, the impact of sodium on immune cells is further accentuated. It has recently been shown that the lesional skin of patients suffering from atopic dermatitis, a Th2-mediated chronic inflammatory skin disease, displays up to 20-fold enriched sodium concentrations compared to those of healthy control skin [20]. Considering that the matched nonlesional skin of these patients displayed sodium concentrations resembling that of skin from healthy individuals excludes a dietary impact or other systemic reasons for this differential distribution of sodium in inflamed versus noninflamed skin within the same patient. Atopic dermatitis is characterized by an epidermal barrier defect that facilitates epidermal water loss. This water loss could potentially be a driving force for sodium enrichment in areas of increased barrier disruption. In addition, dysbiosis with *Staphylococcus aureus* is pathognomonic of atopic dermatitis [30]. *S. aureus* thrives in high salt concentrations at the expense of other species of the commensal skin microbiota [31]. Interestingly, sodium chloride has been shown to drive Th2 cell differentiation and to increase Th2-associated effector functions in memory T cells [20,32]. In particular, IL-4 and IL-13 production are increased upon increased sodium chloride exposure [20]. Together, these effects result in epidermal barrier defects, salt accumulation, dysbiosis and Th2 cell bias in a logical sequence of dysregulated events, ultimately culminating in full-blown eczematous atopic skin lesions [33]. The fibrotic skin in patients with systemic sclerosis [34] and lipedema [35] has also been associated with increased sodium concentrations. Psoriasis, another chronic inflammatory skin disease, in contrast, did not reveal increases in sodium concentrations in lesional or nonlesional skin upon analysis with a highly sensitive and specific neutron activation analysis [20]. This finding indicates that sodium is specifically associated with certain diseases but not a byproduct of chronic inflammation in general.

Interestingly, local infection has also been associated with interstitial sodium accumulation. A subcutaneous injection with Bacille Calmette–Guerin or Freund’s adjuvant in rodents has been found to result in enhanced tissue osmolalities [36]. In humans, superficial streptococcal skin infections have been found to be associated with elevated sodium levels as assessed by ^23^Na-MRI technology [37]. These elevated sodium levels resolved upon antibiotic treatment. Interestingly, this cutaneous sodium accumulation has been shown to promote a macrophage-driven host defense and to strengthen the antimicrobial barrier function [38]. Other organs beyond the skin have also been reported to display elevated sodium concentrations in pathological immune-mediated conditions. Disability and a progressive course in multiple sclerosis have, for example, been associated with sodium accumulation within brain lesions and within the white matter and cortical and deep gray matter [39]. Together, these findings illustrate that the level of sodium deposition is a critical factor for human health and disease through its impact on the immune system.

## 3. The Role of Ionic Signals in Shaping Th1–Th2 Cell Dualism

The discovery of Th1 and Th2 cells in the late eighties sparked the quest for their differentiation and maintenance signals [5,32]. It was only recently that sodium chloride was also noted as a potent inducer of human and murine Th2 cells and a suppressor of Th1 cell responses [20]. Sodium chloride has been shown to increase IL-4 and IL-13 production in T helper cells in a dose-dependent manner and to abrogate IFN-γ production. This effect has been found to be accompanied by the upregulation of the Th2 cell master transcription factor GATA-3 and phosphorylation of its upstream regulator p-STAT6. These effects were orchestrated by the osmosensitive transcription factor nuclear factor of activated T cells 5 (NFAT5), also known as tonicity-responsive enhancer binding protein 5, and its downstream kinase serum- and glucocorticoid-inducible kinase (SGK-1) [20]. Interestingly, this Th2 cell skewing of NaCl can also explain the Th2 bias in atopic dermatitis. The strong enrichment of sodium chloride in the lesional skin of these patients aligns with the preferential enrichment of Th2 cells in atopic skin and the therapeutic benefits of IL-4Ra blockade with monoclonal antibodies. The epithelial sodium sensor Na_x_ has previously been shown to coordinate sodium homeostasis in skin characterized by an atopic barrier defect [40]. Upon the Na_x_ knockdown in vivo, the characteristic features of the eczematous skin resolved [41,42]. Its downstream target, ENaC, a major sodium channel in epithelial cells, is regulated by NFAT5 and SGK-1 [40]. Together, these findings place sodium and its cellular effects into a coherent framework for the pathogenesis of atopic dermatitis.

Dickkopf-1 has been demonstrated to regulate Th2 cell effector functions via the mechanistic target of rapamycin (mTOR) pathway and SGK-1, with implications for *Leishmania major* (*L. major*) infection and house dust mite-induced asthma [43]. Although a direct role of sodium chloride or any other upstream regulator of Dickkopf-1 was not assessed in that study, the involvement of its downstream target SGK-1 suggests that the Th2-regulated disease courses of *L. major* infection and asthma could be sensitive to modifications of the extracellular salt microenvironment. Furthermore, SGK-1 has been shown to be central to mammalian target of rapamycin complex 2 (mTORC2)-driven Th2 cell differentiation since T cell-specific deletion of rapamycin-insensitive companion of target of rapamycin (Rictor), the mTORC2-specific adaptor, abrogated the SGK-1 activation [44].

## 4. The++ Role of Ionic Signals in Th17 Cell Development and Th17 Cell Functions

Th17 cells have gained much prominence as a highly proinflammatory T helper cell subset with pathogenic functions in autoimmune diseases. They are also relevant for the clearance of fungal infections, in particular those caused by *Candida albicans* [45]. Th17 cells display functional heterogeneity. Pro- and anti-inflammatory Th17 cell subsets have previously been identified in both mice and humans [46]. They differ in their expression of IFN-γ and IL-10, respectively [45].

**Figure 2:**
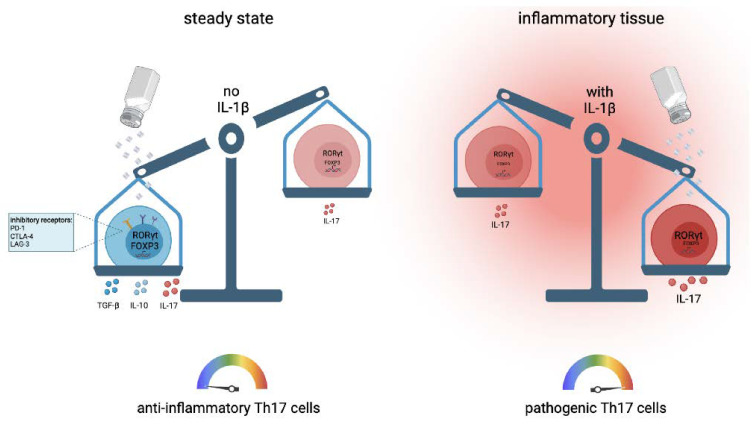
Sodium chloride promotes the anti-inflammatory Th17 cell identity in steady-state conditions but can also enforce Th17 cell pathogenicity in IL-1β enriched microenvironments.

The polarizing cytokines for optimal human Th17 cells are still a matter of debate [46]. Sodium chloride has been demonstrated to exert very strong IL-17 induction in the pioneering work from the Kuchroo and Hafler groups in mice and humans, respectively [47,48]. The IL-17 increase in Th17 cells that are primed from naïve T cell precursors has been shown to require the presence of the Th17-polarizing cytokines IL-1β, IL-6, TGF-β and IL-21 [48]. Fully differentiated memory and effector T helper cells, on the other hand, upregulated the IL-17 expression independent of the polarizing cytokines in the presence of increased sodium chloride concentrations upon T cell receptor stimulation alone [49] (Figure 2). This effect was not only restricted to the CD4^+^ T helper cell lineage, since CD8^+^ cytotoxic T cells were also shown to upregulate IL-17 upon restimulation in high sodium chloride conditions. IFN-γ and IL-4 expression, on the other hand, remained unaffected within the CD8^+^ T cell lineage [20].

Sodium chloride has been shown to act via SGK-1, which turned out to be critical for regulating IL-23R expression, thus stabilizing the Th17 phenotype [47]. Sodium chloride has therefore been proposed to contribute to Th17 cell-mediated autoimmune diseases via the SGK-1–Th17 axis. Interestingly, another mechanism that has been proposed for its role in the pathogenesis of multiple sclerosis is the modulation of the commensal gut microbiota by a high-salt diet [50]. High salt levels have been shown to cause reductions in the abundance of *Lactobacillus* spp., which reverted their Th17 inhibitory functions. Although no direct effects of salt on T cells could be postulated in this particular study, its indirect action on the microbial composition in the gut was ultimately shown to be associated with an autoimmunity.

More recent work demonstrated that sodium chloride, instead, promoted the anti-inflammatory subset of Th17 cells [49] (Figure 2). Sodium chloride upregulated expression of forkhead box P3 (FOXP3), the master regulator of regulatory T (Treg) cells, jointly with IL-17, along with multiple other anti-inflammatory T cell properties, including PD-1, CTLA-4 and Lag3 expression. This effect was also exerted via the NFAT5–SGK-1 signaling axis at high sodium chloride concentrations [49]. This anti-inflammatory Th17 cell phenotype also translated into immunosuppressive functions in vivo, as demonstrated by the reductions in clinical scores after the adoptive transfer of NaCl-stimulated Th17 cells into a mouse model of multiple sclerosis [49]. Together, these results demonstrate an anti-inflammatory impact of NaCl on Th17 cell functionality. This protective effect of NaCl in autoimmunity has recently been further supported with a high-salt diet in a spontaneous mouse model of central nervous system (CNS) autoimmunity [51].

Whether a high-salt diet, which is characteristic of the Western diet of the last 30 or more years, translates into an increased risk for autoimmunity or immunosuppression remains to be seen. In humans, a pilot study demonstrated that a moderate high-salt challenge increased Th17 cell levels via the modulation of the gut microbiota, leading to reduced *Lactobacillus* spp. abundance [50]. Another recent clinical study, however, could not identify any major changes in the immune cell subsets. In particular, Th17 cells were not found to be affected following 2 weeks of high-salt diet consumption [52]. Despite these discrepancies, dietary effects of NaCl on Th17 cell frequencies or functions cannot be entirely excluded with short-term intervention trials, meriting further investigation.

Quantitative evidence for increased sodium chloride concentrations in peripheral tissues in correlation with increased dietary intake is needed in the future to corroborate the causality between salt and immune effects in humans. Furthermore, the mechanistic networks involving the microbiota point to indirect effects of dietary salt on the immune system, suggesting more complex networks by which salt exerts its effects on human health and disease.

## 5. The Role of Ionic Signals in Treg Cells

**Figure 3:**
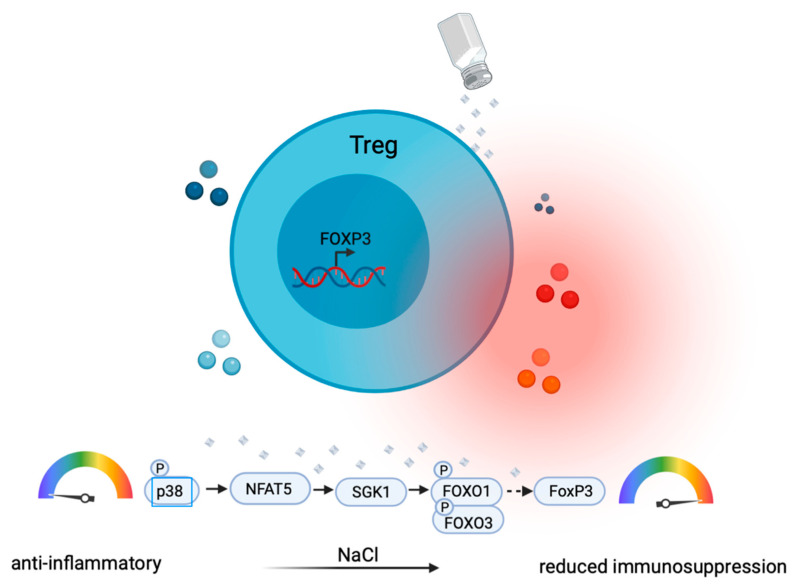
Sodium chloride compromises the anti-inflammatory function of Treg cells by the upregulation of proinflammatory (IFN-γ, IL-17) and downregulation of anti-inflammatory cytokines (IL-10). Sodium chloride leads to phosphorylation of p38 mitogen-activated protein kinase (MAPK), then activation of NFAT5 followed by the upregulation of SGK-1. Forkhead box O1 (FOXO1) and Forkhead box O3 (FOXO3) stabilize the FOXP3 locus, a process, which is compromised by their SGK-1-induced phosphorylation. This promotes the loss of anti-inflammatory Treg functions paired with proinflammatory cytokine production (IL-17, IFN-γ).

Treg cells are professional immunosuppressive T helper cells [53]. They display pronounced plasticity in proinflammatory cytokine microenvironments by the upregulation of IL-17 or IFN-γ, which compromises their anti-inflammatory function [54]. SGK-1, which is induced upon high NaCl exposure, has been shown to not only play a role in promoting Th17 cells (as discussed above), but to also play a critical role in the IL-23R-mediated inhibition of Treg cells [55]. High NaCl concentrations have been shown to impair Treg functions through the upregulation of IFN-γ in Treg cells via the SGK-1 node [56] (Figure 3). In vivo, this translated into impaired Treg functions due to IFN-γ coproduction in a xenogeneic graft-versus-host disease model and in adoptive transfer models of experimental colitis [56]. Cumulatively, these findings suggest that Treg cells are co-opted as culprits in inflammation by losing their Treg identity through the NaCl-mediated upregulation of proinflammatory cytokine production.

Another study did not observe increased IFN-γ expression but rather increased Th17-like differentiation in murine Treg cells upon NaCl treatment [57]. IL-17A^+^ RAR-related orphan receptor gamma T (RORγt)^+^ FOXP3^+^ induced Treg (iTreg) cells were not observed in vitro but were induced in vivo only, suggesting that additional inflammatory signals, such as IL-23 [55] or IL-1β [58], may promote the development of Th17-like Treg cells. Together, these results consistently suggest a proinflammatory conversion of Treg cells into a proinflammatory T cell subset through either IFN-γ or IL-17 induction (Figure 3).

Interestingly, Th17-like Treg cells have previously been shown to adapt to high-salt conditions while maintaining their suppressive functions [57]. Another study supported the functional stability of Treg cells upon high NaCl exposure [59]. The development, differentiation, and functional activities of induced Treg (iTreg) cells were not compromised. Instead, the stability and function of iTreg cells were enhanced in vitro and in vivo. This result contrasted with that of the thymic Treg (tTreg) cells, and this effect has instead been attributed to plasticity in the response to NaCl [59]. These findings highlight the importance of the type of target cell for downstream sodium chloride induced effector functions.

## 6. Regulation of Pro- versus Anti-Inflammatory T Cell Functions by Salt

How can these discrepant findings with respect to pro- versus anti-inflammatory effects of salt on Th17 and Treg cells be reconciled? It has been recently demonstrated for human T cells that the presence of IL-1β exerts a switch factor function for the effects of NaCl [49]. The presence of IL-1β during Th17 cell polarization has been found to be required for NaCl to promote a pathogenic Th17 cell phenotype [49]. This result aligns with the Th17 cell characteristics observed by previous work from the Hafler and Kuchroo laboratories [47,48]. However, according to previous studies, Th17 cell polarization is also feasible in the absence of IL-1β [60,61]. In the absence of IL-1β, sodium chloride has been found to promote an anti-inflammatory Th17 cell identity instead. The differential requirement for IL-1β in the process of Th17 cell priming is contingent on the microbial T cell receptor specificity of the naïve T helper cell [60,61,62]. *C. albicans*-specific Th17 cell differentiation requires IL-1β, whereas *S. aureus*-specific Th17 cell differentiation is feasible in its absence. This differential requirement of proinflammatory IL-1β for Th17 cell priming is due to the production of IL-12 in *C. albicans* but not *S. aureus* co-cultures with antigen-presenting cells. IL-1β proved necessary to compensate for the Th1 bias that would ensue upon IL-12 signaling [60]. The dichotomous outcomes of sodium chloride signaling indicate that the cytokine context determines the overall outcome of sodium chloride effects on T helper cell functions (Figure 2). This finding, therefore, also has implications for the effect of NaCl on T cells with distinct microbial specificities. In line with the differential ability of specific microbes to induce IL-1β production in antigen-presenting cells, NaCl has been shown to increase the anti-inflammatory functions of *S. aureus*-specific Th17 cells but to promote the proinflammatory functions of *C. albicans*-specific Th17 cells [49,60].

## 7. Therapeutic Considerations for Ionic Signaling in T Helper Cells

The crosstalk of cytokines with the ionic salt micromilieu has implications for therapeutic strategies. IL-1β- or IL-1R-blocking agents are already in use in the clinic for the treatment of autoinflammatory and other chronic inflammatory diseases [63]. Therapeutic abrogation of the IL-1 signaling pathway might, in accordance with its switch factor function, promote the anti-inflammatory activities of sodium chloride in hypersalinic tissue microenvironments. Reciprocally, increased IL-1α or IL-1β concentrations, which occur in autoinflammatory syndromes or in chronic inflammation, might enhance the pathogenicity of local salt deposition.

The p38/ mitogen-activated protein kinase (MAPK) pathway, involving NFAT5 and SGK-1, has been shown to regulate FOXP3 and IL-17A expression in human memory T cells under high-NaCl conditions. This pathway could therefore represent an attractive therapeutic target for the modulation of Th17 responses in tissues characterized by a high NaCl content. Likewise, IL-4 and IFN-γ have been shown to be reciprocally regulated by the NFAT5–SGK-1 axis. Mammalian target of rapamycin complex 2 (mTORC2) is an upstream activator of SGK-1 [44]. It has previously been shown to promote Th2 cell lineage differentiation [64]. T cell-specific deletion of Rictor, which is a specific adaptor for mTORC2, has been found to abrogate the activation of SGK-1. This resulted in reduced allergy and increased antiviral immune responses in mouse models. In addition, the Wnt antagonist Dickkopf-1 has been shown to enhance Th2 responses via the mTOR pathway and SGK-1. This affected mite-induced asthma and *L. major* infection in mouse models [43]. Downstream targets of SGK-1 signaling in response to mTORC2 activation are JunB and the long isoform of the transcription factor T cell factor 1 (TCF-1) for Th2 versus Th1 responses, respectively. Overall, the association of mTORC2 signaling with the osmosensitive NFAT5–SGK-1 pathways links salt signaling to mTORC2, thus proposing several downstream signaling molecules as potential therapeutic targets for the modulation of the Th1–Th2 equilibrium.

Additionally, the indirect effects of sodium chloride on the composition of T helper cells and their respective functions can be exerted by the microbiota. The suppressive effect of sodium chloride on *Lactobacillus* spp. has been found to unleash Th17 cell responses through reductions in the levels of tryptophan metabolites, in particular, indole metabolites, with implications for multiple sclerosis [50]. Considering the tremendous impact that has been attributed to the commensal microbiota for a vast array of homeostatic functions as well as diseases [65,66], drug-induced or dietary modulation of its composition is likely to affect a plethora of body functions in complex contextual settings via the indirect actions on the immune system or independent of immune cells. Sodium chloride is also known to affect the endocrine regulatory circuits [67,68]. It downregulates the renin–angiotensin–aldosterone system but upregulates the production of glucocorticoids [69]. These hormonal systems have a direct impact on T cell functions, both pro- and anti-inflammatory [70]. Interestingly, therapeutic interventions can therefore be exerted via the SGK-1 signaling pathway, which is receptive to both sodium chloride and corticosteroids [69,71]. Lastly, the modulation of sodium uptake into peripheral tissues and their long-term deposition might represent target points for therapeutic strategies. Macrophages with their ability to regulate lymph flow, and thus salt clearance through salt sensitive VEGFC, might emerge as therapeutic targets [24]. Since negatively charged macromolecules, such as GAGs, have been postulated to the control overall sodium content, the molecular enzymatic targets responsible for their turnover might correlate with the bioavailability of sodium ions [22]. Despite the lack of solid evidence in humans, the dietary influence on salt mediated immunoregulation should not be discarded before larger studies provide more insights. Overall, the picture emerges that salt signaling in T helper cells can be disrupted for therapeutic purposes on multiple layers and that additional targets will emerge via its indirect effects on systemic regulatory circuits, as well as on the commensal microflora.

## 8. Outlook

Recent studies have pinpointed the strong impact of the ionic signal sodium chloride on the development and function of T helper cells. Although the signaling pathways have been mapped and seem to match to a great extent previously identified osmosensitive regulatory mechanisms, the proximal molecular sensors of sodium chloride remain ill defined. How is salt sensed by a T cell? This question could open up new avenues for therapeutic targeting given the impact of this signal on the regulation of T cell effector functions. Additionally, the crosstalk of salt signaling with other regulatory pathways needs to be further explored. The integration of diverse cellular inputs in conjunction with salt signaling might divert the overall functional response towards a sodium chloride signature. This might partially explain the discrepant cellular outcomes of high sodium chloride treatments within the scientific literature, which is exemplified by IL-1β acting as a switch factor for the dichotomous outcome of the pro- versus anti-inflammatory effects of salt. Furthermore, the specificity of sodium chloride for the reported regulation of T cells needs to be scrutinized. Although sodium chloride represents a physiologically scalable signal, other ionic signals in the tissue microenvironment might exert T cell modulation as well. Potassium, which is enriched in the tumor microenvironment due to the intracellular release from dying cells, represents one example of another potent immune modulator. The elevation of extracellular potassium was shown to impair T cell receptor-driven serin/threonine protein kinase B (also referred to as Akt) -mammalian target of rapamycin pathway (Akt-mTOR) phosphorylation, and thus downstream cytotoxic effector functions leading to CD8^+^ T cell paralysis, which was permissive for tumor growth [72].

Last, the strong impact that ionic signaling exerts on the immune system is not restricted to T cells. Cellular players from both the innate and adaptive immune compartments are likewise exposed to differential concentrations of ionic signals in the tissue microenvironment. While several pathways have been shown to be conserved across cell types, others will be more cell type specific, leaving room for additional salt effects to be observed in the future.

## Data Availability

Not applicable.
